# Preparation and In Vitro Characterization of *Lactococcus lactis*-Loaded Alginate Particles as a Promising Delivery Tool for Periodontal Probiotic Therapy

**DOI:** 10.3390/jfb15050129

**Published:** 2024-05-15

**Authors:** Bettina Wuttke, Katharina Ekat, Oleksandra Chabanovska, Mario Jackszis, Armin Springer, Praveen Vasudevan, Bernd Kreikemeyer, Hermann Lang

**Affiliations:** 1 Department of Operative Dentistry and Periodontology, University Medical Center Rostock, 18057 Rostock, Germany; 2 Institute of Medical Microbiology, Virology and Hygiene, University Medical Center Rostock, 18057 Rostock, Germany; 3 Biomechanics and Implant Technology Research Laboratory, Department of Orthopedics, University Medical Center Rostock, 18057 Rostock, Germany; 4 Medical Biology and Electron Microscopy Centre, University Medical Center Rostock, 18057 Rostock, Germany; armin.springer@med.uni-rostock.de

**Keywords:** periodontal health, probiotic therapy, alginate particles

## Abstract

Probiotic microorganisms are used in a variety of food supplements and medical formulations to promote human health. In periodontal therapy, probiotics are mainly used in the form of gels, tablets or rinses that often tend to leak from the periodontal pocket, resulting in a strongly reduced therapeutic effect. In this pilot in vitro study, we present biodegradable alginate-based particles as an alternative, highly efficient system for a periodontal delivery of probiotic bacteria to the inflammation site. For this purpose, *Lactococcus (L.) lactis* was encapsulated using a standardized pump-controlled extrusion-dripping method. Time-dependent bacterial release in artificial saliva was investigated over 9 days. The effect of freeze drying was explored to ensure long-term storage of *L. lactis*-loaded particles. Additionally, the particles were bound to dentin surface using approved bioadhesives and subjected to shear stress in a hydrodynamic flow chamber that mimics the oral cavity in vitro. Thus, round particles within the range of 0.80–1.75 mm in radius could be produced, whereby the diameter of the dripping tip had the most significant impact on the size. Although both small and large particles demonstrated a similar release trend of *L. lactis*, the release rate was significantly higher in the former. Following lyophilization, particles could restore their original shape within 4 h in artificial saliva; thereby, the bacterial viability was not affected. The attachment strength to dentin intensified by an adhesive could resist forces between 10 and 25 N/m^2^. Full degradation of the particles was observed after 20 days in artificial saliva. Therefore, alginate particles display a valuable probiotic carrier for periodontal applications that have several crucial advantages over existing preparations: a highly stable form, prolonged continuous release of therapeutic bacteria, precise manufacturing according to required dimensions at the application site, strong attachment to the tooth with low risk of dislocation, high biocompatibility and biodegradability.

## 1. Introduction

Periodontitis is a multifactorial, chronic inflammatory disease that develops in association with a dysbiotic biofilm [1]. In this regard, severe periodontitis is the sixth most common disease in the world [2]. The chronic inflammation of periodontal tissues such as periodontal ligament, cementum and alveolar bone is commonly caused by the unbalanced growth of oral polymicrobial pathogens, which can stimulate host defenses and activate proteolytic enzymes, leading to tissue destruction [1,3,4,5,6]. Consequentially, emerging periodontal pockets provide an ideal surface for bacterial colonization and accumulation of pathogenic subgingival plaque that facilitates destructive periodontal inflammation [7]. If left untreated, progressing periodontitis can cause irreversible tissue degradation, resulting in tooth loss [6,7]. Considering the increased incidence in the elderly and the worldwide trend of demographic aging [2], an intensified occurrence of periodontitis and, thus, a greater need for therapy are expected in the future [8].

Unfortunately, insufficient results are often achieved by standard methods of periodontitis treatment using mechanical or chemical disruption of pathogenic biofilm [1,9]. Therefore, antibiotic agents are preferably used in addition to mechanical cleaning in order to further reduce microbiological disbalance [10]. However, antibiotics also impair non-pathogenic microflora. On the other hand, some of the subgingival periodontopathogens are increasingly resistant to common antibiotic drugs. For example, a study by Rams et al. (2014) demonstrated that around 74.2% of examined patients with chronic periodontitis yielded subgingival biofilm resistant to therapeutic concentrations of commonly used antibiotics [11]. These disadvantages demand alternative therapeutic approaches for antibiotic-free restoration of physiological microflora.

Recent research indicated that a highly diverse oral microbiota is critical for periodontal health [12]. A new therapeutic approach suggests that the implementation of probiotic bacteria can additionally be utilized in causal periodontal therapy in order to shift the oral flora towards more mixed species [13,14,15,16,17]. Although its mechanism of action is not yet fully understood, several studies reported positive clinical effects on oral health following probiotic treatment in cases of halitosis [18], fungal diseases [19] and caries [20,21,22]. In fact, probiotics exhibit anti-inflammatory features and promote the expansion of healthy microbiota in impaired periodontium [13,17,23,24,25,26], thereby disrupting the proliferation of pathogenic bacteria and preventing bone degradation [27,28,29]. Clinically demonstrated adjunctive probiotic treatment improved parameters of periodontal health in diseased patients, such as lower plaque and gingival index, reduced bleeding on probing and higher attachment rates [15,29,30,31]. In contrast to other periodontal adjunctive agents, probiotics are advantageous in terms of safety and broad usability among all age groups. Application-related limitations exist only in immunologically susceptible patients [32]. 

*Lactococcus (L.) lactis* in particular is a probiotic species that is known to be beneficial for periodontal health. *L. lactis* is a key microorganism in the dairy industry, producing lactic acid as the main carbohydrate end product. It has been granted “generally recognized as safe” status by the US Food and Drug Administration [33]. Furthermore, *L. lactis* has been added to the European Food Safety Authority’s Qualified Presumption of Safety list, enhancing its versatile beneficial probiotic effects [34]. For example, *L. lactis* demonstrated inhibitory effects on the growth of periodontal pathogens such as *Fusobacterium nucleatum, Porphyromonas gingivalis, Tannerella forsythia* and *Treponema denticola* [35]. In animal models, *L. lactis* protected against bone loss and reduced inflammation in periodontal tissue [36]. These exceptional properties are mostly attributed to the secretion of a specific bacteriocin called nisin, which *L. lactis* is able to produce in significant quantities. Nisin belongs to the class of lantibiotics and is the only bactericide approved by the US Food and Drug Administration and the WHO for use in food preservation. Moreover, nisin can stimulate cells of connective tissue and osteoblasts, thereby promoting regeneration of injured compartments [37]. Additionally, recent studies by Radaic et al. (2022) showed that *L. lactis* significantly reduced the formation and viability of oral pathogenic biofilms in vitro [38,39]. 

In order to achieve a therapeutic effect, probiotic bacteria must be administered in sufficient amounts. However, currently available probiotic formulations for the treatment of oral dysbiosis (chewable tablets, liquid drops and gels) are deficient in terms of prolonged retention of the delivered microorganisms at the inflammation site. Due to the low depot effect, such delivery systems require frequently repeated administration. To this account, polysaccharide-based alginate derived from brown algae is a natural, stable, hydrophilic, mucoadhesive, biodegradable and highly biocompatible substance that has great potential to overcome the shortcomings of conventional oral delivery systems [40].

Due to its physical and biological properties, alginate is widely used in regenerative medicine as a matrix for cell- or bacteria-based applications, in pharmaceuticals for controlled drug release [41], in the food industry for stability enhancement [42,43] and in dentistry for dental impressions [44]. Depending on the intended application, alginate can be cost-effectively produced in different forms, such as fibers, membranes or particles. Indeed, alginate fibers enriched with chlorhexidine or antibiotic agents have been previously described for periodontal therapy [45]. However, little is known about the encapsulation of probiotics in alginate particles for oral application, although such formulations are common for the treatment of gastrointestinal diseases [46]. 

The objective of this present in vitro study was to develop a carrier system for probiotic bacteria with further goals and attributes of (I) easy applicability, (II) stable bacterial release and (III) slow-release kinetics for the bacterial load. We focused on alginate-based particles for such a system as it would ensure a long-acting therapeutic medication in the periodontal pocket. Consequently, we explored different methods for alginate particle preparation loaded with *L. lactis* and investigate its stability as well as the bacterial release behavior in artificial saliva. Furthermore, we evaluated the dentin adhesion of the produced particles using various biodegradable adhesive components. For the first time, we described a promising alginate-based probiotic formulation exclusively for oral application that might be highly beneficial for the treatment of inflammatory periodontal diseases in the future.

## 2. Materials and Methods

### 2.1. Bacterial Strain and Culture

*L. lactis* NZ9000 was provided by the Department of Biochemistry, Groningen Biomolecular Sciences and Biotechnology Institute and Proteomics Centre and Zernike Institute for Advanced Materials, University of Groningen, Groningen, Netherlands [47]. Brain Heart Infusion (BHI) medium (Oxoid, Basingstoke, Hampshire, GB) as a culture medium was prepared at a concentration of 37% *w*/*v* in *Aqua dest*. and autoclaved at 121 °C. A total of 20 mL of BHI was inoculated with cryopreserved *L. lactis* following cultivation under aerobic conditions until its stationary growth phase at 37 °C and 5% CO_2_ overnight. 

### 2.2. Preparation and Loading of Alginate Particle with L. lactis

All procedures were carried out under sterile conditions and all working solutions and liquids were autoclaved. Alginate particles were prepared based on the previously described drip extrusion technique [48,49,50]. Briefly, different concentrations of sodium alginate (1%, 2% or 3%; Sigma-Aldrich, St. Louis, MI, USA) and calcium chloride (1%, 3% or 5%; Sigma-Aldrich) were prepared in 100 mL *Aqua dest.* and left stirring for at least 12 h on a magnetic stirrer (800 rpm).

**Figure 1:**
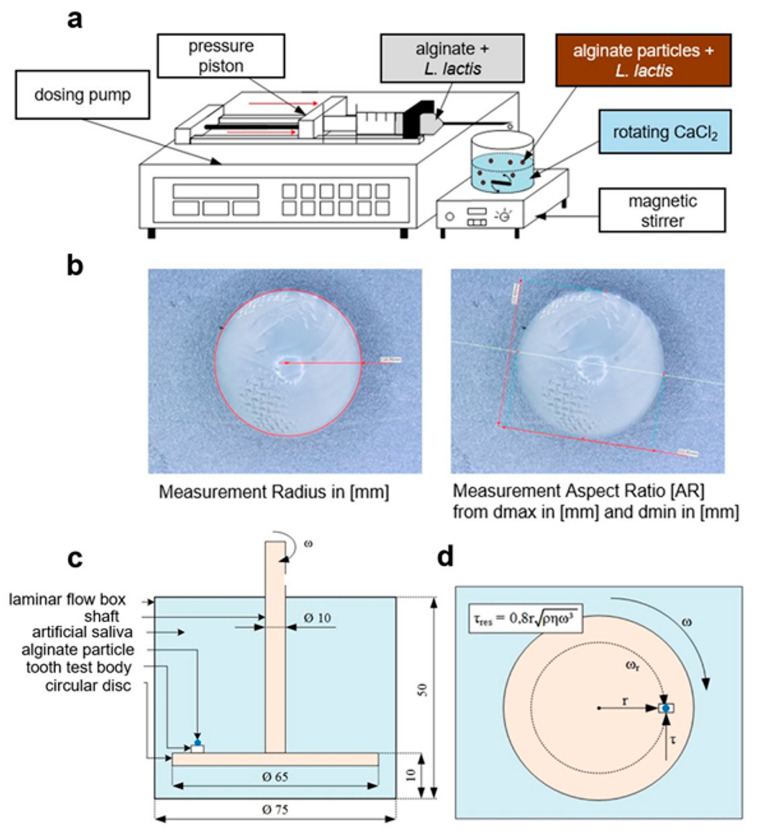
Illustration of methods used to produce and characterize alginate particles. (**a**) Schematic workflow highlights the main technical requirements for production. A dosing pump controlled the pressure piston, which advanced the alginate solution mixed with *L. lactis* through the dripping tip into a rotating CaCl_2_-enriched gelation bath placed on a magnetic stirrer. (**b**) Selected microscopic images exemplify the measurement of size (left) and roundness (right) of the particles in terms of radius (in mm) and aspect ratio (d_max_/d_min_), respectively. Magnification: 100×. (**c**) Schematic set-up of the spinning disk device illustrates the laminar flow chamber filled with artificial saliva that was used to define the bond strength of different adhesives. An alginate particle was adhered to a tooth specimen, which was attached to the circular disk. Rotating shaft translated the increasing angular velocity (ω) until particle detachment. All dimensions in mm. (**d**) Schema represents the calculation formula of the shear stress (τ_RES_), which acts tangentially on the alginate particle.

An overnight culture of *L. Lactis* was centrifuged for 10 min at 2.927× *g* and the pellet was resuspended in 1 mL phosphate-buffered saline (PBS). Subsequently, *L. lactis* suspension was adjusted to an optical density of 0.5 (ca. 10^7^ CFU/mL) at 600 nm (OD600) using a spectrophotometer (SpectraMax M2, Molecular Devices, Ismaning, Germany) and mixed 1:4 with sodium alginate solution (final volume ~5 mL) in a 10 mL disposable syringe (BD Discardit II). A 27 G (Sterican 0.4 mm × 12 mm; B. Braun, Melsungen, Germany) or 20 G cannula (Miraject PL Super 0.9 mm × 22 mm; Hager & Werken, Duisburg, Germany) was attached to the filled syringe. The syringe system was then clamped into a syringe pump device (200 Legacy Syringe Pump, KD Scientific, Holliston, MA, USA; Figure 1a). Different settings summarized in Table 1 were applied to produce uniformly round droplets. The alginate–probiotic mixture was transported dropwise from a distance of 10 cm into a rotating CaCl_2_ solution at a different set rate (60 mL/h or 80 mL/h) and different stirring speeds (100, 400 or 800 rpm). Here, the calcium salt acted as a crosslinker that triggers the gelation of the alginate by exchanging the sodium ions for calcium ions [51]. Alginate particles were formed as soon as the alginate–probiotic drop entered the CaCl_2_ solution. The resulting alginic hydrogel in the form of a particle carried the probiotic bacteria enclosed within its core.

The prepared particles were left in the CaCl_2_ solution for 20 min to allow for solidification. The particles were then collected using an Easystrainer sieve (100 μm, Greiner Bio-One, Frickenhausen, Germany) and subsequently washed twice in 40 mL of autoclaved 0.1 M HEPES buffer (pH = 7.3; Carl Roth, Karlsruhe, Germany) for 10 min. 

### 2.3. Characterization of Alginate Particles in Terms of Size and Shape

The effect of different production settings on size (radius) and shape (sphericity) was evaluated. A total of 16 experimental groups were created based on manipulated manufacturing parameters (Table 1). From each generated group, nine particles from three independent experiments were randomly selected as three technical replicates for the characterization. Microscopic images (VHX 6000, Keyence Deutschland, Neulsenburg, Germany ) were used to measure the radius according to the three-point circle method (Figure 1b, left) and determine the roundness as a ratio of length (maximum diameter, d_max_) to width (minimum diameter, d_min_): AR = d_max_/d_min_ (Figure 1b, right). Thus, the AR of a perfectly spherical particle equals 1.0 [52,53].

### 2.4. Release kinetics of L. lactis and Latex Beads 

The release of *L. lactis* from samples of groups No. 4 (radius 0.99 mm) and No. 16 (radius 1.53 mm; Table 1) into artificial saliva was investigated under aerobic conditions at 37 °C and 5% CO_2_ in a time-dependent manner. Artificial saliva was prepared according to Pratten et al. (1998) using 6 mM NaCl, 2.7 mM KCl, 1.5 mM K_2_HPO_4_ and 3.5 mM KH_2_PO_4_ dissolved in *Aqua dest*. With the addition of 0.1% *w*/*v* Lab Lemco Powder (Oxoid), 0.5% *w*/*v* Proteose Pepton No. 3 (BD Bioscience, Heidelberg, Germany) and 0.2% *w*/*v* yeast extract [54]. pH was adjusted to 6.7. The final solution was autoclaved.

For each experiment, five probiotic-loaded particles were transferred to one well of a 24-well microtiter plate filled with 1 mL artificial saliva and stored under aerobic conditions at 37 °C and 5% CO_2_ over the course of 9 days (1 h, 2 h, 3 h, 4 h, 5 h, 6 h, 24 h, 48 h, 72 h, 144 h, 192 h, 216 h). The artificial saliva was not changed during the test period. At the defined time points, the entire supernatant (1 mL) from one well was transferred to a 1.5 mL reagent vial and vortexed thoroughly. The resulting bacterial suspension was then serially diluted in 1× PBS and 100 µL of each dilution was plated onto BHI agar plates using Eddy Jet Spiral Plater 1.22 (IUL Instruments GmbH, Koenigswinter, Germany). The inoculated plates were incubated at 37 °C and 5% CO_2_ under aerobic conditions for 24 h. The number of colony-forming units (CFU/mL) per dilution stage was then determined and defined as the live bacterial count (LBC) that was released from the particles. The LBC was normalized to one alginate particle. 

Plastic-based inert latex beads (Sigma-Aldrich) with 1 µm in diameter (corresponding to the size of *L. lactis*) were encapsulated in the alginate particles (settings according to group No. 16) and served as a negative control. The incubation was conducted under aerobic conditions at 37 °C and 5% CO_2_. The supernatant containing released beads was analyzed using NucleoCounter^®^ NC-3000 (ChemoMetec A/S, Allerod, Denmark) at the following time points: 2 h, 4 h, 24 h, 48 h, 120 h, 240 h and 360 h.

### 2.5. Qualitative Release Analysis Using Scanning Electron Microscopy (SEM)

The SEM images were used to visualize the release behavior of the internalized bacteria and latex beads at defined time points. For this purpose, the particles were washed twice in 20 mL 0.1 M HEPES for 10 min and fixed in 1 mL of 0.1 M HEPES buffer containing 1% paraformaldehyde and 2.5% (*v*/*v*) glutaraldehyde for 2 h (RT) followed by an overnight incubation at 4 °C. Subsequently, excess fixative was removed by washing twice in *Aqua bidest.* for 10 min (RT). Next, the samples were dehydrated in ethanol in several steps: 10 min incubation in 50% ethanol, 10 min in 90% ethanol and three times every 10 min in 100% ethanol, followed by 2 × 20 min incubation in hexamethyldisilazan (HDMS, Carl Roth, Karlsruhe, Germany). HMDS was aspirated, and the samples were air-dried for 30 min and placed in a desiccator for overnight drying. On the next day, partially cut alginate particles were sputtered with a 10–15 nm thick gold layer using a sputtering device (BalTec AG, Pfäffikon, Switzerland) and examined using a raster electron microscope equipped with a field emission cathode MERLIN^®^ VP Compact (Carl Zeiss, Oberkochen, Germany). Here, the HE-SE2 detector, an accelerating voltage of 5.00 kV and a working distance of approx. 5–6 mm were implemented. Image-relevant parameters such as magnification and µm-scale are stated in the corresponding image caption.

For microscopy of the supernatants, the artificial saliva surrounding the alginate particles was taken and vortexed, and 0.5 mL of each was fixed as described above. A total of 400 µL of the fixed sample was sedimented using a Microstar 17 microcentrifuge (VWR International, Darmstadt, Germany) for 10 min at 3345× *g*, and the pellet was resuspended in 200 µL of 0.1 M sodium phosphate buffer (pH 7.4). A total of 200 µL of the sample was incubated on a poly-L-lysine-coated coverslip (Carl Roth) for 1 h (RT, humid chamber). Excess liquid was aspirated and the coverslips were dehydrated in an ascending acetone series (10 min, 75%; 10 min, 90%; and 2 × 10 min, 100% (*v*/*v*) acetone). Subsequently, the coverslips were dried at the critical point using the EMITECH K850 (Quorum Technologies Ltd., Lewes, UK). The dried samples were sputtered with a 10–15 nm thick gold layer in a vacuum and examined by SEM as described above.

### 2.6. Growth Kinetics of L. lactis in Artificial Saliva

The basal growth kinetics of *L. lactis* in artificial saliva was determined by cultivation of 10^7^ CFU/mL under aerobic conditions at 37 °C and 5% CO_2_ for 9 days. The LBC was defined as described above at the following time points: 1 h, 2 h, 3 h, 4 h, 5 h, 6 h, 24 h, 48 h, 72 h, 144 h, 192 h, 216 h (9 d).

### 2.7. Freeze-Drying and Its Effect on L. lactis Release

Eppendorf tubes filled with five freshly prepared beads were briefly stored on ice in a cryobox and then transferred to liquid nitrogen for 5 min. The particles were cryopreserved at −196 °C. Alpha 1–4 lyophilizer (Christ Laborgeräte, Ostrode, Germany) was used to lyophilize the particles at −40 °C, 1.7 mbar vacuum and 1500 rpm. The resulting lyophilized particles were stored at −20 °C until further use. Subsequently, the particles were rehydrated in water or artificial saliva and incubated at 37 °C and 5% CO_2_ for 1 h, 2 h, 4 h or 24 h. The dehydrated vs. rehydrated states were microscopically evaluated as described above. Briefly, size and shape were determined based on bright-field microscopy (VHX 6000, Keyence Deutschland, Neulsenburg, Germany) and the surface morphology was observed with SEM. The weight was measured using a precision balance (PCE Germany GmbH, Meschede, Germany) to calculate the swelling of the alginate. The *L. lactis* released from a freeze-dried alginate particle (group No. 16) into artificial saliva after 24 h incubation at 37 °C and 5% CO_2_ was quantified as the LBC of the inoculated supernatant on BHI plates.

### 2.8. Dentin Adhesion Tests 

#### 2.8.1. Construction of the Spinning Disk Device 

The dentin adhesion strength was determined using a modified spinning disc device previously reported by Fritsche et al. in 2014. A schematic representation of the experimental setup is summarized in Figure 1c. Additionally, Supplementary Materials Figure S1 provides a general overview of the technical components. A cylindrical liquid container made from translucent Plexiglas (Evonik Industries, Essen, Germany) had an inner diameter of 75 mm. The bottom of the reservoir was glued with Acrifix (Evonik Industries) to an 8 mm thick Plexiglas pane, which in turn was screwed to a 160 mm × 400 mm profile plate (Item Industrietechnik, Solingen, Germany). Thus, the liquid reservoir rested on a Plexiglas base plate. The test chamber was covered by a loosely attached lid made of polyetheretherketone (PEEK), in which a radial shaft seal ran centrally. During the measurement, the lid formed a tight seal of the test chamber. A highly polished circular disc made of coated glass with a diameter of 65 mm was mounted on the stainless-steel shaft with a diameter of 10 mm. The shaft had a thickness of 1 mm and a central hole of 2 cm in diameter, which created a rigid connection to the rotation axis. The stainless-steel shaft ran vertically from an angular gear through an upper and a lower crosshead. The gearbox, in turn, transmitted the rotary motion of the motor (EC-max 40, Maxon Motor, Sachseln, Switzerland) to the shaft. The horizontal lateral bearing of the electric motor enables height adjustment for the motor, gearbox, shaft and specimen. The digital servo amplifier (4-Q-EC, Maxon Motor, Sachseln, Switzerland) was responsible for controlling the electrical motor and monitoring its current speed. The servo amplifier, which was supplied with a current of 5 A and a voltage of 12 V via the EA-PS 3032-05 B laboratory power supply unit (Elektro-Automatik, Viersen, Germany), coordinated the control software (user interface version 1.15; Maxon Motor Control, Maxon Motor, Sachseln, Switzerland) with the electric motor. 

#### 2.8.2. Tooth Specimen Preparation and Experimental Set-Up

This study was approved by the Ethics Committee of the University of Rostock (registration number A 2021-0081, 12 April 2021). The test tooth was acquired during a wisdom teeth surgery. The patient gave informed written consent prior to surgery and sampling according to the Declaration of Helsinki. The test tooth was cleaned with water to remove any tissue or blood residue. A tooth section of 5.5 mm × 3.5 mm with a thickness of 3 mm was prepared using a diamond band saw 300 (Exakt Advanced Technologies, Norderstedt, Germany). The adhesion strength of alginate particles (group No. 16) to the dentin surface was examined using two biodegradable and biocompatible adhesives: Artiss^®^ fibrin glue (Baxter Deutschland, Unterschleißheim, Germany) and Liquiband^®^ Optima skin adhesive (Advanced Medical Solutions, Winsford, UK). Here, the theoretical impact of oral chewing or shearing stress on the adhesive properties was investigated in vitro based on the spinning disk method. The test particles were stained with trypan blue (Deutsche Chemapol, Frankfurt, Germany) for better visualization and glued to the dentin either with 10 µL of Artiss^®^ (5 µL fibrinogen-aprotinin mixed with 5 µL thrombin solution) or 10 µL of Liquiband^®^. The tooth specimen with attached alginate particles was placed in the test chamber and fixed on the circular disc at the radial distance of 2.5 cm from the center (r = 2.5 cm; Figure 1d) using Loctite 4011 glue (Henkel, Düsseldorf, Germany). The chamber was then filled with artificial saliva. The specimen was exposed to different rotation speeds (from 10 rpm to a maximum of 1100 rpm). 

#### 2.8.3. Calculation of the Shear Stress

To assess the adhesion strength, a hydrodynamic shear stress τ_RES_ (force per surface) was calculated (Figure 1d) according to the following equation: $\tau_{RES}=0.8r\sqrt[{}]{{\rho \eta \omega }^{3}}$, where:

- r = 2.5 cm is the radial distance from the particle to the center of the circular disc;
- ρ = 1.0 g/cm^3^ is the density of the artificial saliva that filled the flow chamber (determined using the DSA 5000 M density meter (Anton Paar GmbH, Graz, Austria);
- η = 1.0 mPas is the viscosity of the artificial saliva at 20 °C (determined using Twindrive^®^ Rheometer MCR 702 (Anton Paar GmbH);
- ω is the angular velocity of the circular disc rotating inside the flow chamber calculated from the pre-set speeds (rpm) and the only variable parameter during the measurements: $\omega =\frac{rpm}{60}\times 2\times \pi$.

### 2.9. Statistics

The characterization of the particles was performed with at least three independent biological replicates, including three technical replicates during each experiment, resulting in a total of nine samples (n = 9) per group. For the release studies, technical replicates counted five samples, resulting in fifteen samples (n = 15). The experimental data are presented as mean ± standard deviation (SD). Statistical significance was calculated using a *t*-test or one-way or two-way ANOVA tests with Tukey post hoc correction. The probability levels considered statistically significant were * *p* < 0.05 (significant), ** *p* < 0.01 (very significant), *** *p* < 0.001 (highly significant) and **** *p* < 0.0001 (highly significant). Calculations and graph analysis were performed using Microsoft Excel (Office Professional Plus 2016; Microsoft, Redmond, WA, USA) and GraphPad Prism 8.4.3 (GraphPad Software Inc., Boston, MA, USA).

## 3. Results

### 3.1. Characterization of Alginate Particles by Morphology, Geometry and Weight

Defining the size and form of alginate particles is crucial for future clinical application of alginate particles. Therefore, we varied different parameters of a pump-controlled dripping technique to produce uniformly round *L. lactis*-loaded alginate particles of different sizes.

**Figure 2:**
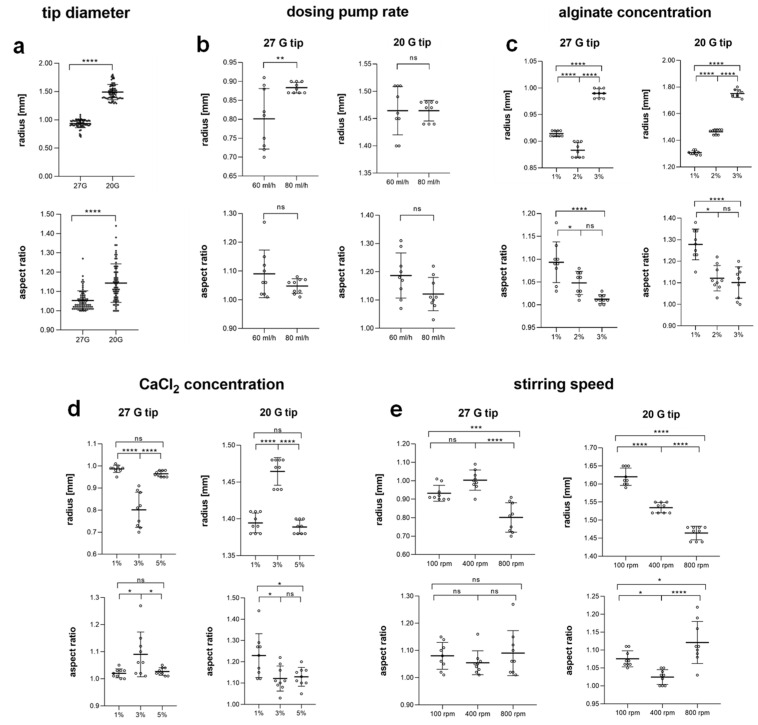
Effect of different production settings on size (radius) and roundness (aspect ratio) of alginate particles. The impact of (**a**) a small (27 G) vs. large (20 G) diameter of the dripping tip, (**b**) dosing pump rate (60 mL/h vs. 80 mL/h), (**c**) alginate concentration (1% vs. 2% vs. 3%), (**d**) calcium chloride concentration (1% vs. 3% vs. 5%) and (**e**) stirring speed (100 vs. 400 vs. 800 rpm) on radius (top) and aspect ratio (bottom) was determined for each sample produced using either a 27 G tip (left) or 20 G tip (right). Data represent mean ± SD of (**a**) n = 72 analyzed particles per condition from 24 independent experiments with three technical replicates per experiment or (**b**–**e**) n = 9 particles per condition from three independent experiments with three technical replicates per experiment (ns = not significant, * *p* < 0.05, ** *p* < 0.01, *** *p* < 0.001 and **** *p* < 0.0001 analyzed with unpaired *t*-test (**a**,**b**) or one-way ANOVA with Tukey post hoc test (**c**–**e**)).

On average, small (0.93 ± 0.07 mm) and mostly round (1.05 ± 0.05) particles were obtained using a 27 G tip, whereas the 20 G tip produced significantly larger (1.49 ± 0.16 mm) and elongated (1.14 ± 0.09) particles (Figure 2a), which were also 4-fold heavier (13.67 ± 0.69 mg) than its smaller counterparts (3.23 ± 0.84 mg; Figure S2). Raising the flow rate of the dosing pump from 60 mL/h to 80 mL/h led to significantly increased size (by 10%; 0.80 ± 0.08 mm to 0.88 ± 0.02 mm, respectively) with no impact on roundness in samples produced by the 27 G tip. Particles produced by the 20 G tip were not affected by increased flow rate (Figure 2b). Highly significant differences in size for both the 27 G and 20 G groups were linked with changing concentration of the alginate solution (Figure 2c). In the group with the 20 G tip, the radius was directly dependent on the alginate solution: increasing concentrations (from 1% to 3%) provoked particle expansion (from 1.31 ± 0.01 mm to 1.75 ± 0.03 mm, respectively). For 27 G tip-derived samples, the middle alginate concentration of 2% produced the smallest particles (0.89 ± 0.01 mm), whereas the 3% solution produced the largest sizes (0.99 ± 0.01 mm). Significantly improved roundness was detected in both groups if the alginate solution was prepared at higher concentrations. Next, we varied CaCl_2_ concentration in the gelation bath (Figure 2d). For both groups, no significant size difference was observed between gelation at low (1%) and high (5%) CaCl_2_ content. However, the use of a 3% CaCl_2_ solution resulted in significantly smaller particles (20% size reduction) in the group with 27 G tips, while significantly larger particles were obtained in the group with 20 G tips. Simultaneously, a higher concentrated gelation bath (3%) significantly reduced sphericity in the 27 G tip group compared to the low (1%) and high (5%) CaCl_2_ content. In contrast, the 20 G tip group demonstrated significantly decreased sphericity at a low CaCl_2_ concentration (1%). Finally, increasing the stirring speed of the gelation bath from 100 rpm to 800 rpm resulted in a significant reduction in particle radius in both the 27 G (by 14%) and 20 G tip groups (by 10%) (Figure 2e). While the sphericity in the first group (27 G) was not affected by the stirring speed, a significant improvement in roundness was observed in the second group (20 G) at the medium stirring speed (400 rpm). 

**Figure 3:**
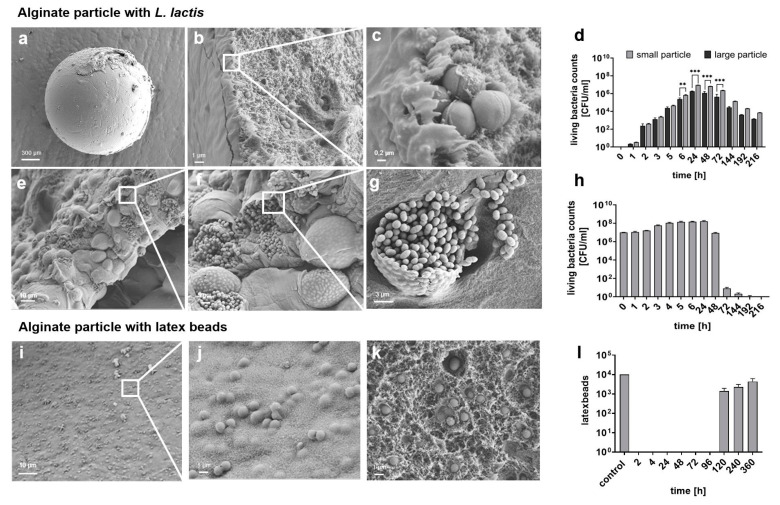
Morphological appearance and release kinetics of *L. lactis* or latex beads enclosed in alginate particle. (**a**) SEM images demonstrate general morphology of freshly prepared alginate particle (magnification 1000×) and (**b**,**c**) *L. lactis* under the surface of a particle after 3 h incubation in artificial saliva (magnification 5000× and 24,000×, respectively). (**d**) The LBC of *L. lactis* released over 216 h from one small vs. one large particle was determined. Data represent mean ± SD of n = 15 (particles) from three independent experiments with five technical replicates (** *p* < 0.01, *** *p* < 0.001 analyzed by two-way ANOVA). (**e**–**g**) The release of *L. lactis* was additionally analyzed by SEM after 24 h incubation in artificial saliva. Magnification: 755× (left), 2350× (middle), 5000× (right). (**h**) Viability of released *L. lactis* in artificial saliva was analyzed over 216 h. Data represent mean ± SD of n = 15 (particles) from three independent experiments with five technical replicates. (**i**–**k**) Surface morphology of freshly prepared alginate particle carrying latex beads was visualized by SEM. Magnification: 1000× (left), 5000× (middle, right). (**l**) Representation of latex bead release from large alginate particle into artificial saliva analyzed over 360 h. Data represent mean ± SD of n = 3 (particles).

Morphologically, particles appeared to be uniformly round with a homogeneous surface as a dense outer layer without adherent microorganisms, as demonstrated by SEM imaging (Figure 3a). A closer look at the surface revealed *L. lactis* embedded within a reticulated scaffold inside the particle (Figure 3b,c).

### 3.2. Investigation of L. lactis Release Kinetics from Alginate Particles

The release of *L. lactis* was examined by LBC released from the spherical particles of the largest (group No. 16; 1.53 mm in radius) and the smallest (group No. 4; 0.99 mm in radius) over the course of 9 days (216 h). These two experimental groups were selected based on the highest reproducibility of the size and roundness across the groups of smaller (No. 1 to No. 8) or larger particles (No. 9 to No. 16; Figure S3). The total *L. lactis* quantity enclosed within one large particle (from group No. 16) was determined as 1.44 × 10^5^ ± 2.09 × 10^4^ CFU/mL (Figure S4). This value served as a control. During the first 6 h, the released bacterial amount from a large particle constantly increased by a factor of 10^2^, peaking at 24 h with 1.82 × 10^6^ ± 1.08 × 10^5^ CFU/mL (Figure 3d), thus significantly exceeding the initially encapsulated bacterial quantity (*p* < 0.01, Figure S4). After this point, bacterial release decreased continuously. Similar kinetics were observed for smaller particles. However, the released bacterial amount from smaller particles significantly exceeded the LBC detected for larger particles by a 2-fold factor at the observation time point of 6 h, and by a 5-fold factor at the observation time points between 24 h, 48 h and 72 h. Active bacterial growth within a particle was confirmed using SEM imaging (Figure 3e–g). It was observed that large accumulations of bacteria formed and spread, which finally led to the rupture of the outer alginate shell and the release of *L. lactis*. 

### 3.3. Examination of L. lactis Growth Kinetics in Artificial Saliva

The determined LBC of the released *L. lactis* could be affected by its additional growth in artificial saliva. To exclude this potential variable from the actual released quantity, the LBC of *L. lactis* cultured in artificial saliva was determined at the corresponding time points between 0 h and 216 h (Figure 3h). At the starting point (0 h), 9.91 × 10^6^ ± 9.56 × 10^5^ CFU/mL were detected. The highest number of viable *L. lactis* was observed after 24 h of cultivation (1.64 × 108 ± 8.58 × 106 CFU/mL), followed by a decrease to baseline levels after 48 h. No vital bacteria were detected after 216 h of cultivation. 

### 3.4. Evaluation of Particle Stability 

To assess the baseline stability of the alginate particles, inert latex beads of the same size as *L. lactis* served as a negative control and were incorporated into the particles instead of *L. lactis*. The morphological appearance of such particles was visualized using SEM imaging, demonstrating small irregular bumps all over the surface (Figure 3i). These were caused by latex beads embedded just below the surface (Figure 3j). Similarly to *L. lactis*-loaded particles, latex beads showed uneven distribution within the net-like framework (Figure 3k).

The release of latex beads from an alginate particle (group No. 16) was analyzed analogously to *L. lactis*-loaded samples. Initially, 1 × 10^4^ units of latex beads were encapsulated (Figure 3l). During the first 4 days, no latex beads could be observed in the artificial saliva. On the fifth day (120 h), 13.9% of the initial load was detected (1.39 × 10^3^ ± 5.52 × 10^2^ units). This amount doubled over the next 5 days (240 h) and represented 22.6% (2.26 × 10^3^ ± 8.43 × 10^2^ units) of the control. A total of 43.7% of the control (4.37 × 10^3^ ± 1.83 × 10^3^ units) was released after 15 days (360 h). Additionally, digital microscopic analysis demonstrated a slight enlargement of the alginate particles during the first 5 days of incubation at 37 °C in artificial saliva (Figure S5a,b). The dimensional stability lasted for approx. 10 days and substantially decreased after this time point (Figure S5c,d). On day 20, the particles appeared to be vastly degraded, showing visible decay products (Figure S5e).

### 3.5. Analysis of Main Particle Features after Freeze-Drying 

**Figure 4:**
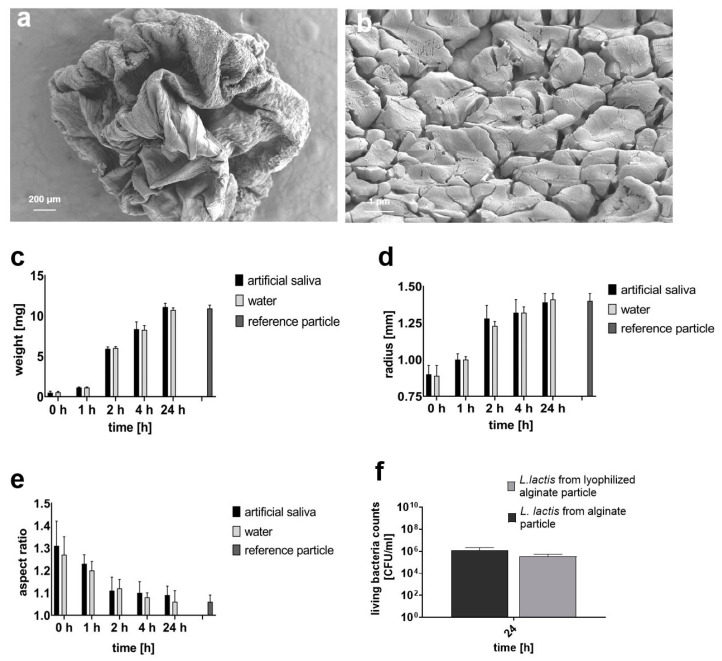
The effect of freeze-drying on particle morphology, weight, size, roundness and bacterial release. (**a**) SEM image displays a freeze-dried alginate particle. Magnification: 41×. (**b**) Enlarged SEM image of the particle surface after freeze-drying. Magnification: 10,000×. Following rehydration in artificial saliva or water for 24 h, the particle (**c**) weight, (**d**) radius and aspect ratio (**e**) were evaluated and compared to a non-dried reference particle. Data represent mean ± SD of n = 5 (particles) from three independent experiments with two or three technical replicates. (**f**) Comparison of LBC of *L. lactis* released from a non-lyophilized vs. lyophilized particle after 24 h incubation in saliva. Data represent mean ± SD of (**c**–**e**) n = 9 (particles) or (**f**) n = 15 (particles) as three or five technical replicates from three independent experiments, respectively.

The *L. lactis*-loaded particles were freeze-dried to investigate their stability after long-term storage. Subsequently, the viability of *L. lactis* was examined after rehydration of freeze-dried particles. Morphologically, the freeze-dried particles shrank to about one-third of their original size, resulting in solid, folded particles of about 1 mm in size, with an angular shape (Figure S6) and a rough porous surface, as illustrated by SEM images (Figure 4a,b). 

The rehydration was carried out either in water or in artificial saliva. Parameters of weight, radius and AR were examined over time. Within the first 1 h of incubation in artificial saliva, the weight increased from 0.49 ± 0.19 mg to 1.13 ± 0.06 mg and quintupled to 5.92 ± 0.22 mg after an additional 1 h of incubation. The maximum weight was reached at 24 h (11.09 ± 0.46 mg) and was comparable to the non-dried particles (reference). No differences in weight were observed between incubation in saliva or water (Figure 4c). A positive trend was also revealed for parameters of radius and AR. After 4 h of incubation, the rehydrated particles reached 94.2% of the reference radius (1.32 ± 0.09 mm in saliva and 1.40 ± 0.05 mm in water, respectively) reaching nearly 100% of the reference radius after 24 h (1.39 ± 0.06 mm in saliva and 1.41 ± 0.04 mm in water) (Figure 4d). Considering roundness, the AR value decreased by 26% within the first 2 h to 1.11 ± 0.06 for saliva-stored and to 1.12 ± 0.04 for water-stored particles (Figure 4e). Using water as a medium resulted in 100% rehydration at 24 h (1.06 ± 0.05 for water and 1.06 ± 0.03 for reference). Saliva rehydration achieved an AR of 1.09 ± 0.04 at the same time point. Additionally, digital microscopy images illustrate the gradual rehydration process (Figure S7). Furthermore, the LBC of *L. lactis* released from non-dried particles after 24 h incubation in artificial saliva was comparable to the bacterial count from a rehydrated particle (1.82 × 10^6^ ± 1.08 × 10^5^ CFU/mL vs. 3.67 × 10^5^ ± 9.25 × 10^4^ CFU/mL, respectively; Figure 4f).

### 3.6. Dentin Adhesion Tests

**Figure 5:**
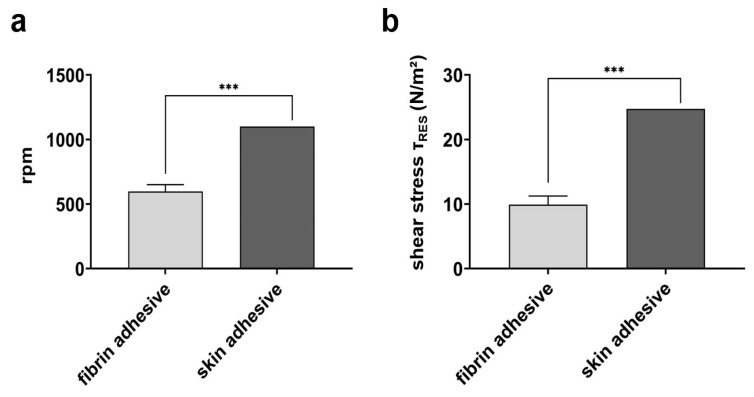
Analysis of the detachment of alginate particles fixed with fibrin vs. skin adhesive from the dentin surface. (**a**) The maximum rotation speed (rpm) leading to the detachment of particles from the dentin was determined. (**b**) Calculated shear stress (τ_RES_) that led to a particle detachment. The values represent mean ± SD for n = 9 (particles) analyzed using an unpaired *t*-test (*** *p* < 0.001).

To estimate the theoretical resistance of a particle to the possible mechanical displacement caused by oral forces acting on it, a sample from group No. 16 was adhered to the dentin surface using biodegradable fibrin or skin adhesive and examined by using the spinning disk principle. This technique allows for an in vitro simulation of oral shear stress exertion on alginate particles as a function of the rotation speed (in rpm). A maximum speed of 597.78 ± 52.6 rpm causing particle detachment from the dentin surface was measured for fibrin adhesive and corresponded to a shear stress of 9.93 ± 1.3 N/m^2^. In contrast, the skin adhesive achieved a significantly stronger attachment as the maximum speed was measured at a doubled rate (1100 ± 0.0 rpm), resulting in a shear stress of 24.73 ± 0.0 N/m^2^ (Figure 5a,b).

## 4. Discussion

Alginate can serve as a binding matrix for different chemical compounds or microorganisms, whereby its production and utilization widely vary depending on the desired effect. In gastroenterology, the encapsulation of bacterial matter in alginate-based polymers is a cost-effective and favorable technique to ensure successful probiotic therapy [46]. In periodontology, alginate-based systems are predominantly known for the administration of anti-inflammatory [55,56] or antibacterial agents [57], while the oral administration of probiotics for periodontal therapy remains under-researched. In this study, we present a method for the preparation and subsequent in vitro characterization of alginate particles loaded with *L. lactis* probiotics for future therapeutic in vivo applications. Round alginate particles could be reproduced uniformly within a radius range between 0.80 and 1.75 mm. The analyzed kinetics of *L. lactis* release demonstrated that the release rate was significantly higher in smaller particles than in larger particles. In both cases, however, the highest effective amount of released bacteria (10^6^ CFU/mL) was reached after 24 h of exposure to artificial saliva. Furthermore, we observed almost complete recovery of the size and shape of freeze-dried particles within the first 4 h of rehydration in artificial saliva, while the viability of *L. lactis* was not affected by freeze-drying. In general, the *L. lactis* particles were stable under physiological conditions for about 10 days and showed a high degree of degradation by day 20. Additionally, the adhesion of alginate particles to the dentin surface was facilitated by biodegradable adhesives, whereby a highly stable bond was demonstrated.

Pump-controlled extrusion dripping is a common method for the production of spherical hydrogel particles, enabling simultaneous encapsulation of desired substances [48,50,58,59]. Variation of different parameters during the production process usually results in a different shape and size of the end product. In general, the round shape is the most favorable for the encapsulation of target compounds, as it provides a larger surface area relative to the volume. This allows for an even and effective distribution of the active agent in the surrounding medium, leading to a prolonged and controlled release of the enclosed substance [60]. Weight and size also portray important specifications for clinical application in terms of appropriate dimensioning for the successful treatment of periodontal defects. The gelatin-based periodontal drug delivery system Periochip^®^ (Dexcel-Pharma, Alzenau, Germany), for example, has a fixed size of 4 mm × 5 mm × 0.35 mm and weighs 6.9 mg [61]. Other products, such as Bio-Gide^®^ collagen membranes (Geistlich, Baden-Baden, Germany), offer larger products (min. 13 mm × 25 mm, 100 mg) that have to be manually adjusted prior to the application. In contrast, alginate particles can be produced easily, time-efficiently and cost-efficiently and without highly specialized technical equipment on site in the required dimensions. Based on pocket measurements or radiographs, the desired particle size could be predefined to fit into any periodontal pocket deeper than 2 mm (Figure S8). Noteworthy, the low weight (range of 3.25–13.66 mg) could facilitate therapy by minimizing the patient’s perception of the particles. Although particles smaller than 0.1 mm could also be produced (i.e., microparticles) [46], their retention within a gingival pocket might be insufficient due to a high chance of slippage caused by sulcus fluid [62,63,64]. Therefore, using particles within the mm range appears advantageous over their micro-counterparts. 

In accordance with previous reports [65], our study demonstrated that the cannula diameter (27 G vs. 20 G) had the most significant influence on the particle radius. Moreover, the morphology of the particles was also rounder when a smaller cannula was used. This effect is probably attributed to the relaxation time of the droplet once it hits the gelation bath. A larger diameter of the dripping tip therefore transmits a larger volume to the droplet, disturbing the reshaping of the particle due to the increased relaxation time [50]. Increasing the dosing rate and concentration of the alginate solution also had a positive effect on the size and improved spherical properties. Other parameters such as the stirring rate in a gelation bath or its concentration can also affect the shape of the end product [50]. In general, it is recommended to adjust the rotation speed to the speed of the particle formation. However, stirring too slowly could lead to interactions between the particles that are forming on the same point on the surface of the gelation bath. On the other hand, too high agitation may develop strong centrifugal forces, resulting in the deformation of the particles. In this study, we examined several settings of stirring rates (100 rpm, 400 rpm and 800 rpm). As a result, the most optimal sphericity was achieved at medium speed (400 rpm). Considering the preparation of the gelation bath, higher-concentrated solutions often enhance the spherical shape and contribute to a uniform size featuring a more homogeneous network, as stated in several reports [66]. However, a direct correlation between calcium concentration and particle morphology was not evident in our study. Finally, the optimum conditions for the production of smaller particles (0.99 mm) were observed for 27 G tip, 60 mL/h dosing rate, 800 rpm stirring rate, 3% alginate and 3% CaCl_2_ solution (group No. 4). If larger particles (1.53 mm) are required, pre-setting a 20 G tip, an 80 mL/h dosing rate, a 400 rpm stirring rate and a 2% alginate and 3% CaCl_2_ solution (group No. 16) are recommended.

Furthermore, we investigated the integrity and durability of the unloaded particles incubated in artificial saliva for 15 days. Studies on the stability and degradation of alginate particles usually use conventional drying and weighing techniques to monitor the change in morphology [67,68,69]. To our best knowledge, however, there are only a few studies on non-contact monitoring of swelling and degradation directly in a solution without any manipulation of the particles [68]. In this study, a non-contact, digital microscopy-based analysis was performed to evaluate the stability. In general, an initial swelling of the particles is often observed. Passive absorption of the surrounding fluid leads to gradual expansion while maintaining an elastic 3D structure [70,71], which could be advantageous for flexible adaptation in the periodontal defect. In this present work, the alginate particles doubled in size by day 5 and remained stable up to day 10. Starting from day 15 onwards, progressive disintegration became apparent. By day 20, the original shape was completely dissolved, leaving diffuse disintegration products of former particles. In the oral cavity, the durability might be reduced due to additional dynamic forces [72,73]. Yet, it is possible to extend the rate of degradation by incorporating additional cross-linking agents into the gelation bath [74] if a higher durability is required.

Previously, *L. lactis* released from alginate particles was examined under simulated gastrointestinal conditions [75] or in the context of milk fermentation [76,77]. However, probiotic alginate particles have not yet been evaluated in a periodontal context using artificial saliva. In this study, we examined the release kinetics of *L. lactis* from alginate particles of different sizes represented by groups No. 4 (small; 0.99 mm) and No. 16 (large; 1.53 mm). In both groups, the minimum therapeutic quantity of 10^6^ CFU/mL [78,79,80] was achieved after 24 h incubation in artificial saliva that lasted for about 48 h. Noteworthily, the amount of released bacteria was significantly higher for the smaller particles than for the larger particles, achieving 5-fold greater kinetics. In fact, small particles have a higher surface-to-volume ratio than large particles [81], resulting in greater distribution of bacteria over a larger surface area and therefore faster release. Subsequently, we examined the basal viability of the non-encapsulated *L. lactis* in artificial saliva. The assessed living bacterial counts revealed a non-significant increase in bacterial growth. However, a vast decline of viable bacteria was detected after three days of cultivation in artificial saliva. On day 9 (216 h) of cultivation in artificial saliva, the presence of vital bacteria could no longer be detected. In contrast, loaded particles demonstrated a residual release between 10^3^ and 10^4^ CFU/mL at day nine. Previously, several groups observed a similar effect of a higher bacterial survival rate encapsulated in alginate compared to the viability of carrier-free bacteria [46,82,83]. There is evidence that encapsulated *L. lactis* might utilize algin molecules [84,85] or traces of bacterial growth medium [86,87] as an energy source for replication. On this account, our SEM analysis additionally demonstrated expanding bacterial colonies forming nest-like niches underneath the surface of the particles [84,88]. Further expansion led to a rupture on the surface, followed by an escape of the bacteria into the surrounding artificial saliva. Furthermore, particles with enclosed latex beads (amorphous polymers) did not show any rupture of the alginate surface or a release of the embedded compounds within the first three days of incubation in artificial saliva. However, the beads started to release from the particle in constant proportion (14% of the total amount) beginning from day five to fifteen, which is probably due to the decomposition of the unloaded particle initiating at the same time point. Therefore, the bacterial release from the particle is an active, growth-based and biodegradation-dependent process that is limited by the surface-to-volume ratio of the particle and the nutritional mass provided by the algin substrate.

Regarding utility in everyday dental practice, the freeze-drying of particles would be a suitable method to optimize the transport, storage and preservation of probiotics encapsulated in alginate particles [83,89]. Freeze-dried alginate particles resemble granules, similar to the granular bone substitutes for use in regenerative dentistry [90,91]. After freeze-drying, the particles shrink to about a third of their original size; the shape changes to a hailstone-like, irregular pattern; and overall general solidification occurs. These effects were also previously described by other groups [92]. However, the freeze-dried particles can be easily rehydrated in an appropriate solution [87]. We demonstrated that a complete recovery of shape, size and weight was achieved after incubation for 4 h in water or artificial saliva. Thus, if placed in a periodontal pocket, the freeze-dried particles would regain their original morphology by absorbing the sulcus fluid or saliva. Indeed, Silberberg (1989) and Ullah et al. (2022) highlighted that hydrogels possess “memory” properties, allowing for a return to the original reference configuration even after prolonged deformation, such as freeze-drying [53,93]. Additionally, the progressive expansion of the particles could limit undesired slippage in the pocket. Despite the increased porosity of the surface as visualized by SEM, the amount of released bacteria after 24 h in artificial saliva was comparable to the non-dried particles, which was also observed by Mirtič et al. (2018) [87]. Although some researchers stated that freeze-drying resulted in significantly lower survival rates of *L. lactis* [92], no loss of bacterial activity was detected in our work. The viability and functionality of immobilized probiotics could remain stable even after several months of storage [92], which is particularly important with regard to flexible application in everyday dental practice. 

To ensure maximum therapeutic effect, the alginate particles must remain in the periodontal pocket until their function as a probiotic carrier is fulfilled [94,95,96]. In principle, the mechanical stability of alginate particles was described as sufficient if no excessive forces were applied [97]. Moreover, in contrast to Periochip^®^ or other conventional dental drug carriers, alginate particles have a customizable shape and mucoadhesive hydrophilic properties [98,99,100,101]. However, oral forces such as sulcus or inflammation-related fluid flow [102], or additional mechanical stress, could disrupt the local stability. Therefore, we facilitated the adhesion of a particle to the dentin surface using a commercial biodegradable adhesive from the class of skin (Liquiband^®^) or fibrin (Artiss^®^) adhesives and subsequently evaluated the bond strength in a modified flow chamber described by Fritsche et al. (2014) [103]. This in vitro system has so far made it possible to quantitatively analyze the adhesion of bone cells to implant surfaces. Similarly, a tooth specimen carrying a particle was attached to the spinning disk that simulated the chewing and shearing stress of the oral cavity. As a result, the skin adhesive provided twice as strong of a hold as the fibrin adhesive: in the first case, a shear stress of ~25 N/m^2^ was required to detach the particle, while the latter did not tolerate forces exceeding 10 N/m^2^. Although it is still difficult to assume the average shear stress acting in the human periodontium, many researchers have performed in vitro experiments using fluid flow in the range of 0.1–2 N/m^2^ to investigate the effect of shear stress on isolated dental cells [104]. Based on this scope, adhered alginate particles can resist the commonly assumed hydrodynamic forces and are therefore highly suitable for oral application. In addition, the use of bioadhesives to improve particle retention at the target site would be clinically unproblematic, as these compounds are already widely used in routine clinical practice [105,106,107]. For example, fibrin adhesives are well tolerated and have long been used in various fields of medicine (i.e., cardiac and neurosurgery), including dentistry for wound sealing and tissue repair [106,108,109]. Fibrin adhesives were first used in periodontal surgery to treat recession with fixed gingival grafts [110]. Furthermore, fibrin sealants are currently the subject of clinical research to clarify whether fibrin glue can be used as an alternative to sutures in periodontal surgery [111]. Other advantages in addition to the adhesive strength involve faster wound healing, lower infection rates, full resorption and high biocompatibility [105,108,109,112]. Noteworthily, recent studies have implemented fibrin adhesives as carriers for the controlled release of antibiotics and growth factors, as well as carrier materials for the construction of (stem) cells, cartilage and tendon tissue [113,114,115]. Therefore, fibrin adhesives might be highly beneficial for facilitated attachment of the particles in the potentially inflamed or bleeding periodontal pocket. In contrast, the topical skin adhesive Liquiband^®^ Optima is a commonly used synthetic adhesive system for sealing external wounds (skin incisions, abrasions, grazes) on the scalp, chin and face [116,117] and degrades in vivo after 21 days. Despite the higher adhesive strength of a skin adhesive, fibrin adhesives may be more favorable to use due to their natural composition, which reduces the risks of allergic reactions or intolerances [109,112]. In terms of administration, both systems allow for simple handling. The fibrin adhesive Artiss^®^ can be applied as a spray to the tooth surface in the periodontal pocket, which is particularly suitable for areas that are difficult to access. The winged applicator of the LiquiBand^®^ Optima skin adhesive enables safe, controlled and highly precise application. 

In a clinical context, the efficacy of local adjuvant probiotic therapy has been widely demonstrated in vivo [13,17,26,118]. Generally, the minimum number of viable cells that must be present in products containing probiotic microorganisms, as demonstrated in human clinical trials, is estimated to be between 10^6^ and 10^8^ CFU per gram (CFU/g) of the final product or 10^8^ to 10^10^ CFU/day (for 100 g or 100 mL of ingested food) [119,120]. Alginate particle offers at least three days of therapeutic release of *L. lactis* within the range of 10^6^ CFU/mL at the desired application site. Additionally, the highly stable lyophilized form guarantees simple storage and transport options. The non-toxic, inexpensive and easy-to-manufacture alginate particles also meet biocompatibility and biodegradation requirements [121]. Furthermore, the presented system requires less patient compliance than other forms that involve multiple administrations in gel or tablet form. In terms of safety, *L. lactis* is considered a rare human pathogen, and cases of *L. lactis* infections are not frequently reported. Certain clinical isolates of *L. lactis* that contain virulence genes can pose a health risk to patients with underlying diseases, although they are generally harmless to healthy people [122]. *L. lactis* infections in humans usually cause clinical symptoms such as cholecystitis, endocarditis, arthritis and septicemia [123]. Therefore, it is important to distinguish between pathogenic and non-pathogenic strains before administering *L. lactis* as a probiotic product.

Several limiting factors should be acknowledged. This study was conducted in vitro and does not include in vivo verification. Therefore, further in vivo investigation is necessary to confirm the therapeutic efficacy of the suggested probiotic formulation. The particle preparation technique should be optimized to enhance morphological reproducibility. Additional experiments concerning bacterial growth by fermentation of alginate inside the alginate beads have to be conducted [84,88,124]. More detailed investigations are needed to determine the extent to which encapsulated *L. lactis* bacteria are able to exert a positive influence on the oral microbiome by, e.g., bacteriocin production [125]. For example, studies should be carried out on oral multi-species biofilms [120,126]. Another point to be addressed in subsequent studies is to what extent the alginate particles and their degradation products could serve as a scaffold for the formation of further biofilms by, e.g., pathogenic microorganisms. Furthermore, the adhesive strength of a particle to the dentin surface was facilitated using bioadhesive compounds and examined in a specialized in vitro flow chamber. Cementum was not considered for the adhesion analysis as it did not provide sufficient area in the tooth specimen for particle attachment (cementum layer is 0.02–0.2 µm thick [127], while dentin offers > 1.5 mm thickness [128]). In addition, the bond strength to cementum would probably not differ significantly from that of dentin, as the chemical composition of these two regions is similar (around 60% inorganic apatite and 40% collagen in dentin [129] vs. 65% apatite minerals, 23% organic matrix and 12% water in cementum [130]). The in vitro adhesion analysis indicated the maximum resistance strength to the external dynamic forces. However, the outcome might differ in vivo. Additionally, future studies should re-evaluate the particle retention in the periodontal pocket with or without the use of bioadhesives and examine the potential immunogenicity of the particles as a foreign body. Finally, additional in vitro and in vivo testing with bioadhesives is pivotal to exclude any effect on the bacterial release, viability and function. 

## 5. Conclusions

We presented a method for (I) production, (II) characterization and (III) storage of an alginate-based probiotic delivery system. Different manufacturing process parameters allowed for the control of particle size and roundness. Both small and large alginate particles released *L. lactis* in therapeutic quantities for up to three days. This considerably exceeds the therapeutic duration of most contemporary probiotic products for periodontal treatment. Long-term storage in a freeze-dried formulation did not affect the stability and functionality of the particles or the enclosed bacteria. Particles naturally decomposed in artificial saliva within a period of 20 days. Although additional in vivo validation is required in future studies, the alginate-based system is a convenient probiotic bacterial carrier system with promising prospectives for clinical application in dentistry.

## 6. Patents

The described production process for probiotic-loaded alginate particles has been patented as a “depot system for dental applications containing probiotics” (DE102018126394A1) [131].

## Figures and Tables

**Table 1:** Overview of the sample groups defined by different technical settings. Settings required for the production of the smallest roundest (group No. 4; 27 G tip; 0.99 mm in radius) or the biggest roundest (group No. 16; 20 G tip; 1.53 mm in radius) particles are underlined. Abbrev.: C = concentration, No. = number.

Cannula	Manipulated Parameters	Group No.	Pump Rate	C (Alginate)	C (CaCl_2_)	Stirring Speed
27 G tip	pump rate	1	60 mL/h	2%	3%	800 rpm
		2	80 mL/h	2%	3%	800 rpm
	C (alginate)	3	60 mL/h	1%	3%	800 rpm
		2	60 mL/h	2%	3%	800 rpm
		4	60 mL/h	3%	3%	800 rpm
	C (CaCl_2_)	5	60 mL/h	2%	1%	800 rpm
		1	60 mL/h	2%	3%	800 rpm
		6	60 mL/h	2%	5%	800 rpm
	stirring speed	7	60 mL/h	2%	3%	100 rpm
		8	60 mL/h	2%	3%	400 rpm
		1	60 mL/h	2%	3%	800 rpm
20 G tip	pump rate	9	60 mL/h	2%	3%	800 rpm
		10	80 mL/h	2%	3%	800 rpm
	C (alginate)	11	80 mL/h	1%	3%	800 rpm
		10	80 mL/h	2%	3%	800 rpm
		12	80 mL/h	3%	3%	800 rpm
	C (CaCl_2_)	13	80 mL/h	2%	1%	800 rpm
		10	80 mL/h	2%	3%	800 rpm
		14	80 mL/h	2%	5%	800 rpm
	stirring speed	15	80 mL/h	2%	3%	100 rpm
		16	80 mL/h	2%	3%	400 rpm
		10	80 mL/h	2%	3%	800 rpm

## Data Availability

The original contributions presented in the study are included in the article and Supplementary Materials, further inquiries can be directed to the corresponding author.

## Supplementary Materials

The following supporting information can be downloaded at: https://www.mdpi.com/article/10.3390/jfb15050129/s1, Figure S1: Construction of the spinning disk device; Figure S2: Effect of the different dripping tip size (27 G vs. 20 G) on the weight of alginate particles; Figure S3: Sample distribution across analyzed groups in terms of size (radius) and roundness (aspect ratio) of alginate particles; Figure S4: Comparison of released amount of *L. lactis* from a large particle to initially encapsulated quantity; Figure S5: Digital microscopic analysis of the stability of alginate particle in artificial saliva; Figure S6: Digital microscopic analysis of lyophilized vs. non-lyophilized particle; Figure S7: Digital microscopic analysis of the swelling behavior of lyophilized alginate particles; Figure S8: Digital microscopic image exemplifying future clinical application of an alginate particle.
