# Supplementary material for: Preparation and In Vitro Characterization of Lactococcus lactis-Loaded Alginate Particles as a Promising Delivery Tool for Periodontal Probiotic Therapy
Source: J Funct Biomater. 2024 May 15;15(5):129. doi: 10.3390/jfb15050129 (PMC11121860; doi:10.3390/jfb15050129)
Supplement: Supplementary file 1 [file jfb-15-00129-s001.zip › jfb-2928488-supplementary.pdf]

Journal of Functional Biomaterials

Supplementary Material

Preparation and In Vitro Characterization of *Lactococcus lactis*-Loaded Alginate Particles as a Promising Delivery Tool for Periodontal Probiotic Therapy

Bettina Wuttke [1,†], Katharina Ekat [2,†], Oleksandra Chabanovska [1,*,†], Mario Jackszis [3], Armin Springer [4], Praveen Vasudevan [1], Bernd Kreikemeyer [2] and Hermann Lang [1]

1 Department of Operative Dentistry and Periodontology, University Medical Center Rostock, 18057 Rostock, Germany
2 Institute of Medical Microbiology, Virology and Hygiene, University Medical Center Rostock, 18057 Rostock, Germany
3 Biomechanics and Implant Technology Research Laboratory, Department of Orthopedics, University Medical Center Rostock, 18057 Rostock, Germany
4 Medical Biology and Electron Microscopy Centre, University Medical Center Rostock, 18057 Rostock, Germany; armin.springer@med.uni-rostock.de
* Correspondence: oleksandra.chabanovska@med.uni-rostock.de (O.C.)
† These authors contributed equally to this work.

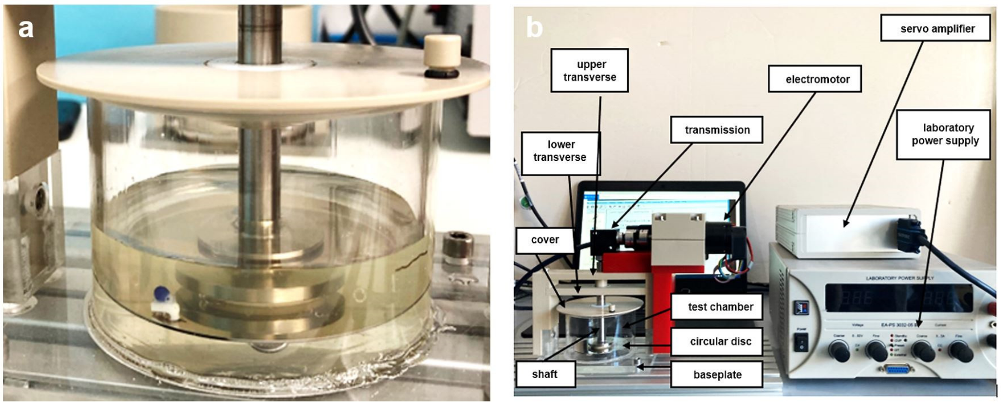

Figure S1. Construction of the spinning disk device. (**a**) Image demonstrates the laminar flow box filled with artificial saliva. The alginate particle is stained blue and attached to a tooth specimen on a circular disk. (**b**) Technical components for the *in vitro* measurement of the shear force.

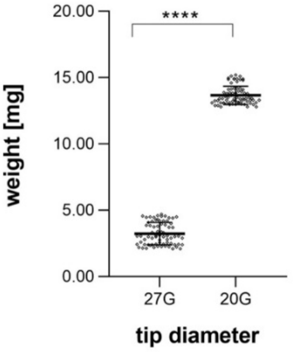

Figure S2. Effect of the different dripping tip size (27G vs. 20G) on the weight of alginate particles. Data represent mean ± SD of n = 72 analyzed particles per condition from 24 independent experiments with 3 technical replicates per experiment (****$p < 0.0001$ analyzed with unpaired t-test).

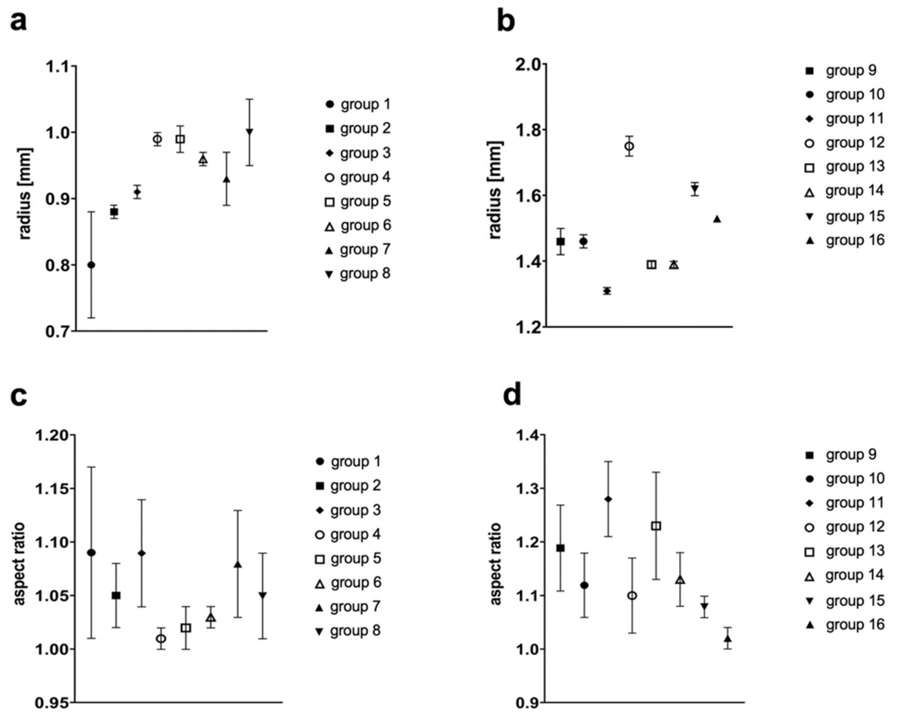

Figure S3. Sample distribution across analyzed groups in terms of size (radius) and roundness (aspect ratio) of alginate particles. (**a**) The technical pre-settings applied to groups No. 1 to 8 produced particles under 1.1 mm in radius, whereas settings for (**b**) groups No. 9 to 16 produced particles larger than 1.2 mm. (**c**,**d**) Representation of the effect of the different pre-settings on the aspect ratio in each group. Data shown as mean ± SD of n = 9 particles per condition from 3 independent experiments with 3 technical replicates per experiment.

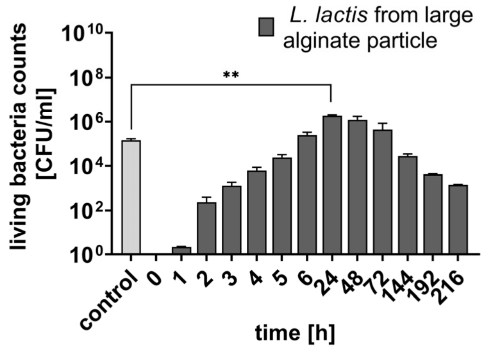

Figure S4. Comparison of released amount of *L. lactis* from a large particle to initially encapsulated quantity. Data represent mean ± SD of n = 15 analyzed particles per condition from 3 independent experiments with 5 technical replicates per experiment (** $p < 0.01$ analyzed with unpaired t-test).

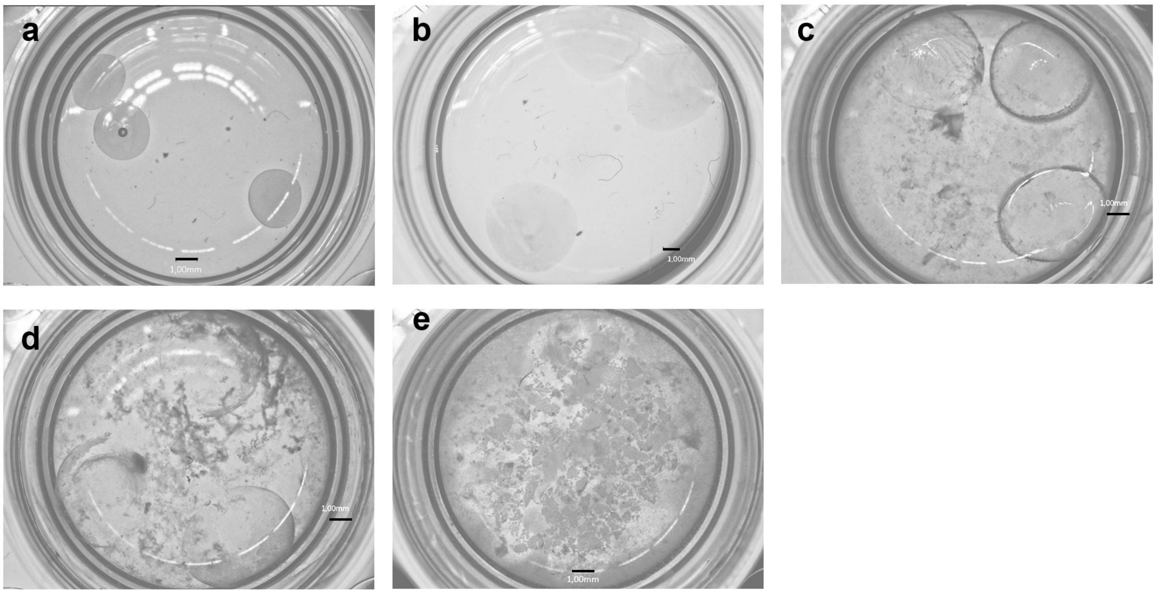

Figure S5. Digital microscopic analysis of the stability of alginate particle in artificial saliva. (**a**) Microscopic image of n = 3 alginate particles (group No. 16) directly after insertion into artificial saliva. (**b**) Particles appeared considerably enlarged after 5 days incubation. (**c**) Incipient degradation was detected after 10 days incubation. (**d**) 15 days of incubation led to visible disintegration of particles. (**e**) Particles were fully dissolved after 20 days incubation in artificial saliva. Magnification: 20×.

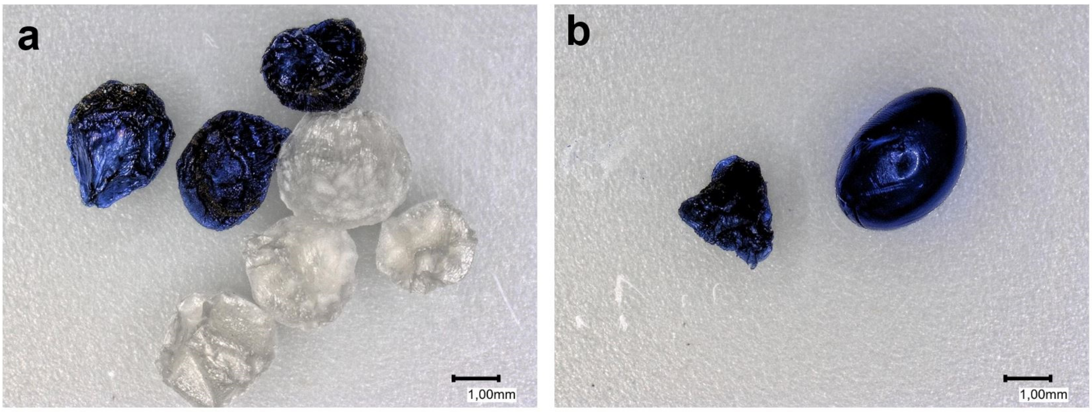

Figure S6. Digital microscopic analysis of lyophilized vs. non-lyophilized particle. (**a**) Lyophilized particles, three of them stained in trypan blue for an optimal visualization. Magnification: 30x. (**b**) Lyophilized particle (left) in comparison to non-lyophilized reference particle (right). Magnification: 30×.

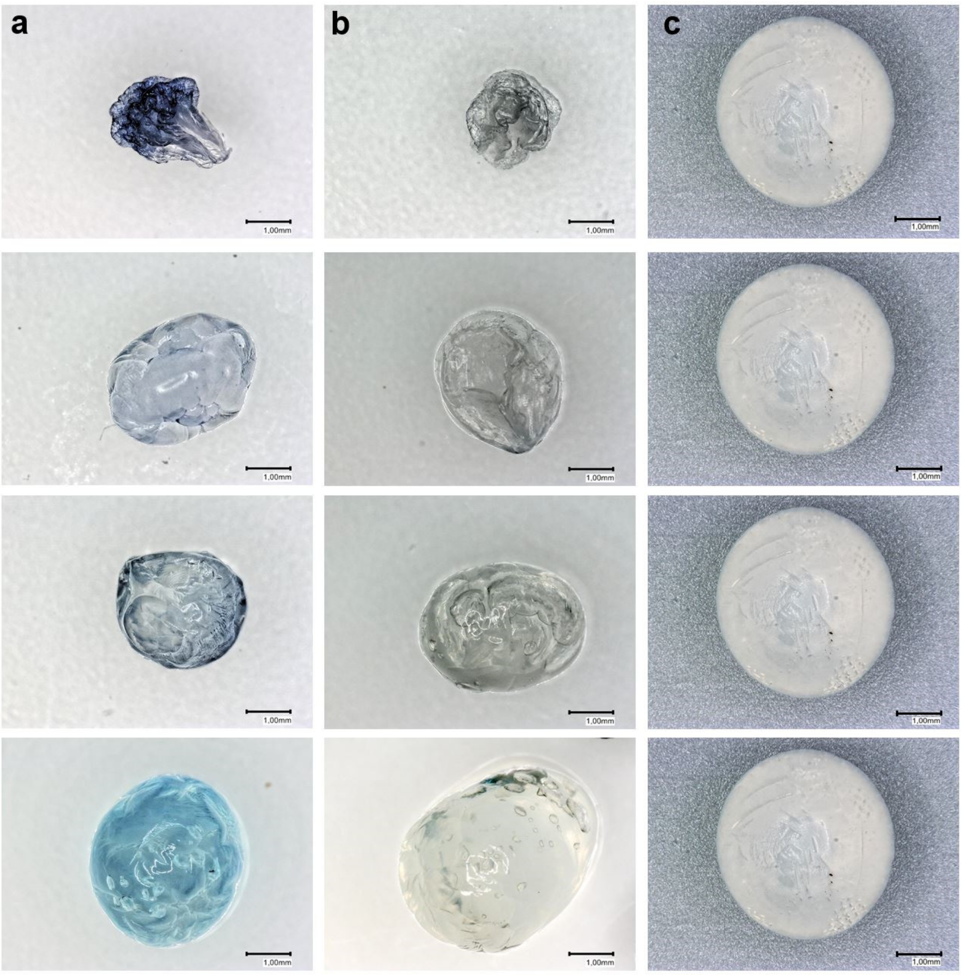

Figure S7. Digital microscopic analysis of the swelling behavior of lyophilized alginate particles. Lyophilized particles were rehydrated in (**a**) artificial saliva or (**b**) water and compared to (**c**) non-lyophilized reference particles. Images were taken after 1 h, 2 h, 4 h and 24 h of incubation. Scala = 1 mm. Magnification: 50×.

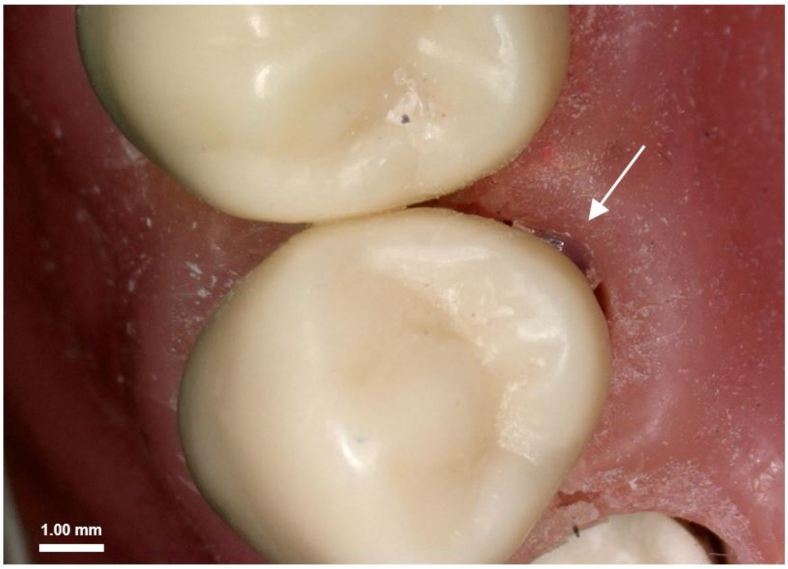

Figure S8. Digital microscopic image exemplifying future clinical application of an alginate particle. An alginate particle loaded with *L. lactis* is placed in a lingual periodontal pocket (tooth 44) on a phantom model of the human jaw. Magnification: 10×.
